# Collagen Type 1 Accelerates Healing of Ruptured Fetal Membranes

**DOI:** 10.1038/s41598-017-18787-9

**Published:** 2018-01-12

**Authors:** Haruta Mogami, Annavarapu Hari Kishore, R. Ann Word

**Affiliations:** Department of Obstetrics and Gynecology, Green Center for Reproductive Biological Sciences, University of Texas Southwestern Medical Center, Dallas, Texas USA

## Abstract

Preterm premature rupture of membranes (pPROM) is a major cause of preterm birth. Recently, extracellular matrix-directed treatment is applied for wound healing. Here, we used a pregnant mouse model to test the efficacy of collagen type 1 gel for healing of the prematurely ruptured fetal membranes. Although injection of PBS into the ruptured fetal membranes resulted in 40% closure, injection of collagen type 1 improved closure rates to 90% within 72 h. Macrophages of the M2 wound healing phenotype were entrapped in the collagen layer. In primary human amnion mesenchymal cells, collagen type 1 gels activated collagen receptor discoidin domain receptor 2 (DDR2) to induce myosin light chain phosphorylation and migration of injured amnion mesenchymal cells. These findings define the mechanisms for matrix-directed therapeutics for pPROM.

## Introduction

Preterm labor is the leading cause of perinatal morbidity and mortality^1^. Preterm premature rupture of membrane (pPROM) is defined as the rupture of membrane occurring before 37 weeks of gestation, which is associated with 30–40% of preterm deliveries and occurs in approximately 1–3% of all pregnancies^2^. In contrast to rapid advancements in the field of perinatal medicine, there is no effective treatment of pPROM. Current treatment of pPROM is limited to expectant management with antibiotics and corticosteroids, and use of tocolytic agents is controversial^3^. Approximately 30% cases of pPROM are infection-related requiring immediate intervention (delivery) for fear of infection to fetus (fetal inflammatory syndrome) and maternal sepsis. However, the remaining pPROM cases are unrelated to infection but may be associated with smoking, low body mass-index, maternal stress, and intrauterine bleeding as well as iatrogenic pPROM caused by amniocentesis or fetoscopy. In most pregnancies complicated by pPROM, premature birth is inevitable because labor ensues spontaneously within days. A small proportion, however, remain undelivered^2^ with unexpected spontaneous sealing of the ruptured membrane^4^. For the treatment of noninfectious pPROM, we may assist the healing of fetal membrane by augmenting natural capacity of regeneration of fetal tissue.

Previously, we developed a model of sterile rupture of the fetal membranes in mice^5^ demonstrating that ~60% of ruptured fetal membranes with a 20 g needle do not heal within 72 h despite recruitment of fetal macrophages to the site of injury and epithelial-mesenchymal transition (EMT) of amnion epithelial cells to close the wound. Recently, extracellular matrix-directed treatment has been developed in wound healing, and the role of matricellular signaling is increasingly recognized^6^.

Using our mouse pPROM model, we found that injection of collagen gel into the ruptured site dramatically accelerated closure rates of amnion from 40 to 90%. This accelerated healing was accompanied by entrapment of arginase-1-positive macrophages and enhanced migration of amnion mesenchymal cells mediated by activation of motor protein, myosin, via collagen receptor, discoidin domain receptor 2 (DDR2).

## Results

### Collagen gel accelerated healing of ruptured membranes

**Figure 1:**
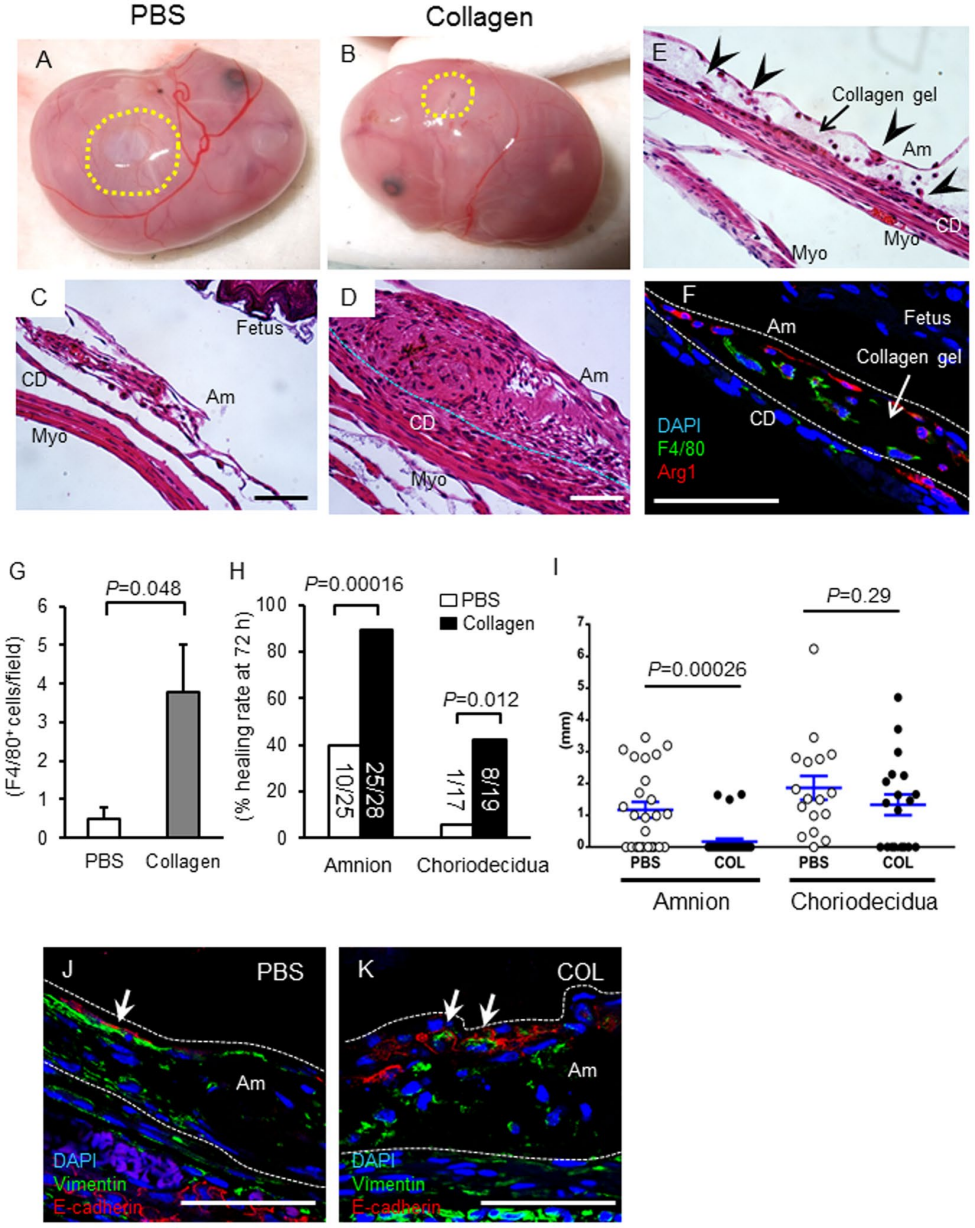
Collagen gel accelerated healing of ruptured fetal membranes *in vivo*. (**A** and **B**) Macroscopic view of ruptured membrane at 72 h. Yellow dotted circle indicates ruptured site. PBS (A) or collagen (B) injection. (**C** and **D**) Hematoxylin and eosin (H&E) staining of ruptured site at 72 h. PBS (C) or collagen (D) injection. Bars, 50 μm. (**E**) H&E staining of collagen-injected fetal membrane close to ruptured site at 72 h. Note that collagen gel layer was formed between amnion and choriodecidua (arrow) and immune cells were trapped inside the gel (arrow head). (**F**) Immunofluorescence staining for F4/80 (green), Arg1 (red) and DAPI (blue) in the collagen layer at 48 h. Note that F4/80-Arg1-double positive macrophages were trapped inside the collagen gel. Arg1 was also expressed in amnion epithelial cells. Bar, 50 µm. (**G**) Number of F4/80 macrophages at the ruptured site between amnion and choriodiecidua (i ncluding collagen layer) per field at 24 h. n = 4–6 in each group. (**H**) Percent closure of ruptured amnion or choriodecidua at 72 h. Number of completely closed ruptures/total ruptures is indicated by the bar. P value, χ^2^. n = 17–28 punctures from 8 pregnant mice in each group. (**I**) Diameter of ruptured membrane sites in amnion and choriodecidua at 72 h of puncture. Each symbol represents one rupture. Blue bar indicates mean and SEM. n = 17–28 punctures from 8 pregnant mice in each group. (**J** and **K**) Immunofluorescence staining for vimentin (green), E-cadherin (red) and DAPI (blue) at healing site of amnion injected with PBS or collagen at 48 h. Note that vimentin-positive cells were observed in the epithelial layer of amnion in both groups (arrow). Bars, 50 µm.

Previously, we described a mouse model of sterile ruptured fetal membranes using 26 or 20 g needles^5^. In this large rupture model, only 40% of ruptured healed spontaneously 72 h after rupture. In this study, we tested the efficacy of collagen gels *in vivo* using our pPROM mouse model. Fetal membranes were ruptured with a 20 G needle (ø 0.91 mm, one puncture per gestational sac), and 20 µl of type 1 collagen gel (2 mg/ml) or phosphate buffered saline (PBS) was injected at the ruptured site between myometrium and fetal membranes. Excess gel did not leak into the amniotic cavity (confirmed by histology). Similar to previous results, 72 h after PBS injection, ~1/2 of puncture wounds remained visible (Fig. 1A) and not healed at the microscopic level (Fig. 1C). Collagen injection, however, resulted in almost complete healing of the ruptured membranes both by gross examination (Fig. 1B) and at the microscopic level (Fig. 1D). Collagen-induced healing was accompanied by increased amnion thickness from aggressive proliferation and migration of amnion mesenchymal cells (Fig. 1D). Importantly, a collagen layer was observed between amnion and the underlying choriodecidua (Fig. 1E). Unexpectedly, numerous arginase 1 (Arg1)-positive M2-phenotype (wound healing type) macrophages were trapped in this collagen layer (Fig. 1F). Collagen gel trapped more macrophages between the amnion and choriodecidua than PBS (Fig. 1G). Importantly, complete closure rate of amnion was over 90% with collagen compared with only 40% with PBS, (Fig. 1H) and average wound diameter decreased significantly at 72 h (from 1.17 ± 0.25 to 0.17 ± 0.10 mm, P = 0.00026, Fig. 1I).

Although healing rates of choriodecidua were less than that of amnion, collagen increased the closure rate of choriodecidua at 72 h (42% compared with 6% for PBS) (Fig. 1H). Wound diameters of choriodecidua were not statistically different (Fig. 1I). EMT-like cells (E-cadherin and vimentin double-positive) were observed at the surface of healing amnion in both PBS- and collagen- injected membrane (Fig. 1J and K). The number of vimentin/E-cadherin cells in ruptured site of amnion was asimilar in both groups (0.3 ± 0.1, collagen versus 0.5 ± 0.1 per HPF, PBS n = 4–6 in each group). It should be noted that although healing of amnion was accelerated by collagen, amniotic fluid volume did not improve substantially (3.3 ± 0.6 µl in rupture with PBS, and 3.3 ± 0.6 µl in rupture with PBS, and 5.3 ± 1.0 µl in rupture with collagen, compared with physiological levels of 60–80 µl).

### Collagen gel stimulated migration of human amnion mesenchymal cells

**Figure 2:**
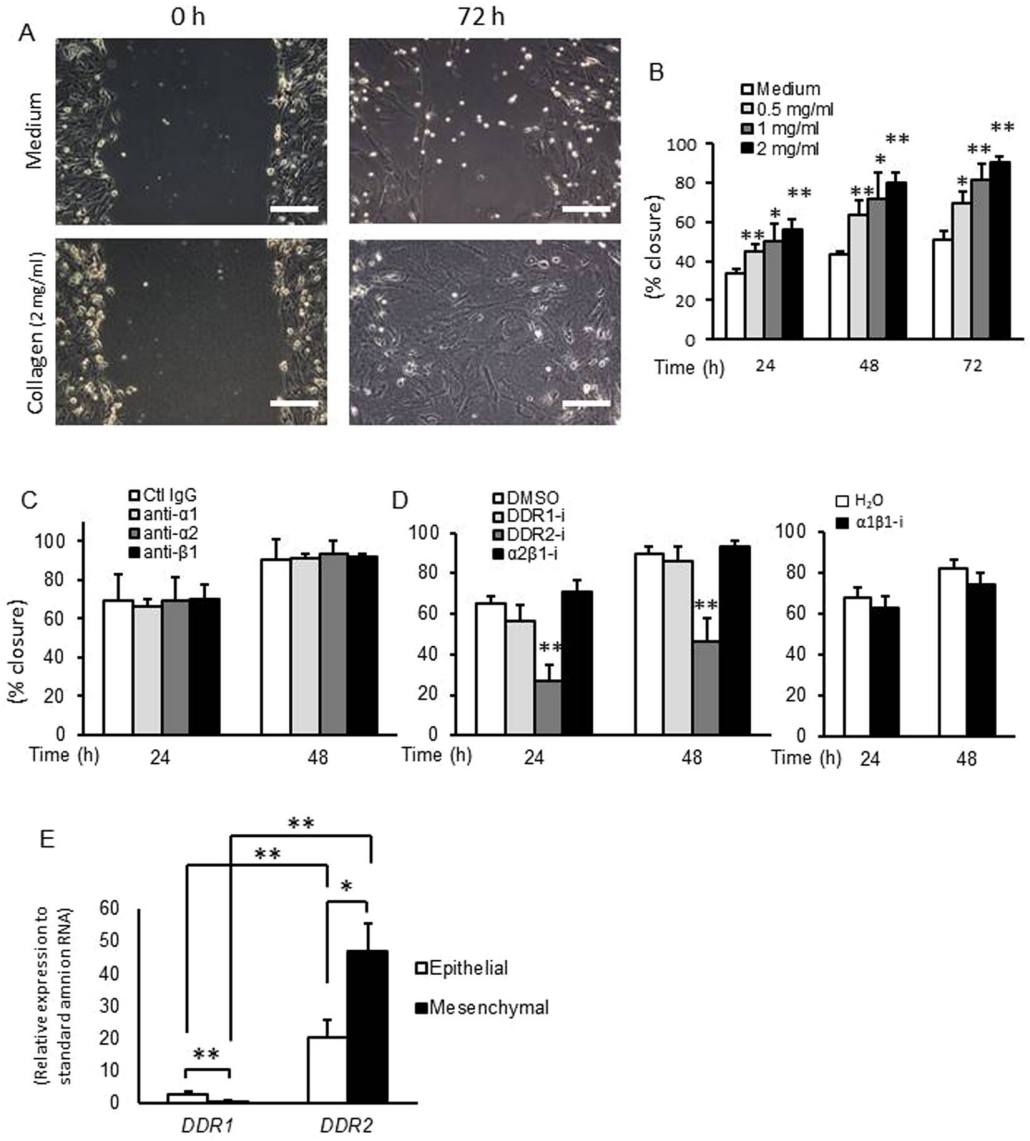
Collagen gel stimulated migration of human amnion cells. (**A** and **B**) Wound healing scratch assay of primary human amnion mesenchymal cells with collagen gel. (**A**) Representative images at 0 and 72 h. Bars, 200 µm. (**B**) Percent closure of scratched area of amnion mesenchymal cells treated with different concentration of collagen gel. (**C**) Wound scratch assay with integrin blocking antibodies. Scratched amnion mesenchymal cells were covered by collagen gel including 10 μg/ml of α1, α2, or β1 integrin blocking antibodies or control IgG. After solidification of gel, serum free medium containing same concentration (10 μg/ml) of blocking antibodies or control IgG was overlaid. (**D**) Wound scratch assay with inhibitors of α1β1 and α1β2 integrin, DDR1, and DDR2. Scratched cells were treated with collagen gel including 1 μM inhibitor (α1β1, α2β1 integrin, DDR1, DDR2) or vehicle (DMSO or water). Serum free medium including same concentration (1 μM) of inhibitors were overlaid. (**E**) DDR1 and DDR2 mRNA expression in human amnion epithelial and mesenchymal cells. Gene expression was normalized to that of GAPDH. The value of each gene was compared by including cDNA standard samples in the same assay plate (standard curve method). Error bars represent SD. n = 3 in each group. *P < 0.05, **P < 0.01.

Next we investigated the mechanisms by which collagen gel stimulated healing of amnion using primary human amnion cells. To test the potential of type 1 collagen to enhance amnion cell migration, monolayer culture of human primary amnion epithelial or mesenchymal cells were seeded on non-coated plastic plates. At confluency, cells were scratched and type 1 collagen gel or medium alone was immediately overlaid. Type 1 collagen dramatically stimulated migration of mesenchymal cells compared with medium-only (Fig. 2A), and this effect was dose-dependent (Fig. 2B). As collagen concentrations increases, gels become stiffer accompanied by an increased rate of migration of mesenchymal cells. Closure of non-covered cells (medium-only) was barely 50% at 72 h whereas mesenchymal cells covered by 2 mg/ml of collagen reached 90% wound closure. Interestingly, collagen-induced migration was limited to mesenchymal cells with no effect on injured amnion epithelial cells (Figure S2A).

Extracellular matrix proteins, including fibrillary collagens, stimulate cell behavior through cell surface receptors, including integrins (especially α1β1 and α2β1 integrins) and the known collagen receptors discoidin domain receptors (DDRs, DDR1 and DDR2)^7^. These receptors regulate a wide range of cell behavior including cell adhesion and migration. Blocking antibodies for α1, α2, and β1 integrins did not inhibit collagen-induced migration of mesenchymal cells (Fig. 2C). Next, DDR1 inhibitor (DDR1-IN-1), DDR2 inhibitor (LCB 03-0110), inhibitors of integrin α1β1 (Obtustatin) and α2β1 (BTT 3033) were tested (Fig. 2D). Of these, only DDR2 inhibitors blocked collagen-induced migration of mesenchymal cells (Fig. 2D). Using qPCR and standard curves, *DDR2* mRNA levels were 85-fold that of *DDR1* in mesenchymal cells (Fig. 2E). In epithelial cells, DDR2 expression was also predominant but less than that of mesenchymal cells (Fig. 2E). siRNA-mediated knockdown of DDR2 in mesenchymal cells revealed 50–60% reduction of *DDR2* mRNA and protein (Figure S2B, S2C). Similar to DDR2 inhibitors, knock-down of DDR2 inhibited collagen-induced migration of amnion mesenchymal cells significantly (Figure S2D). Collectively these data suggest that type 1 collagen not only provides a scaffold for migration but stimulates migration of human amnion mesenchymal cells through the collagen receptor, DDR2.

**Figure 3:**
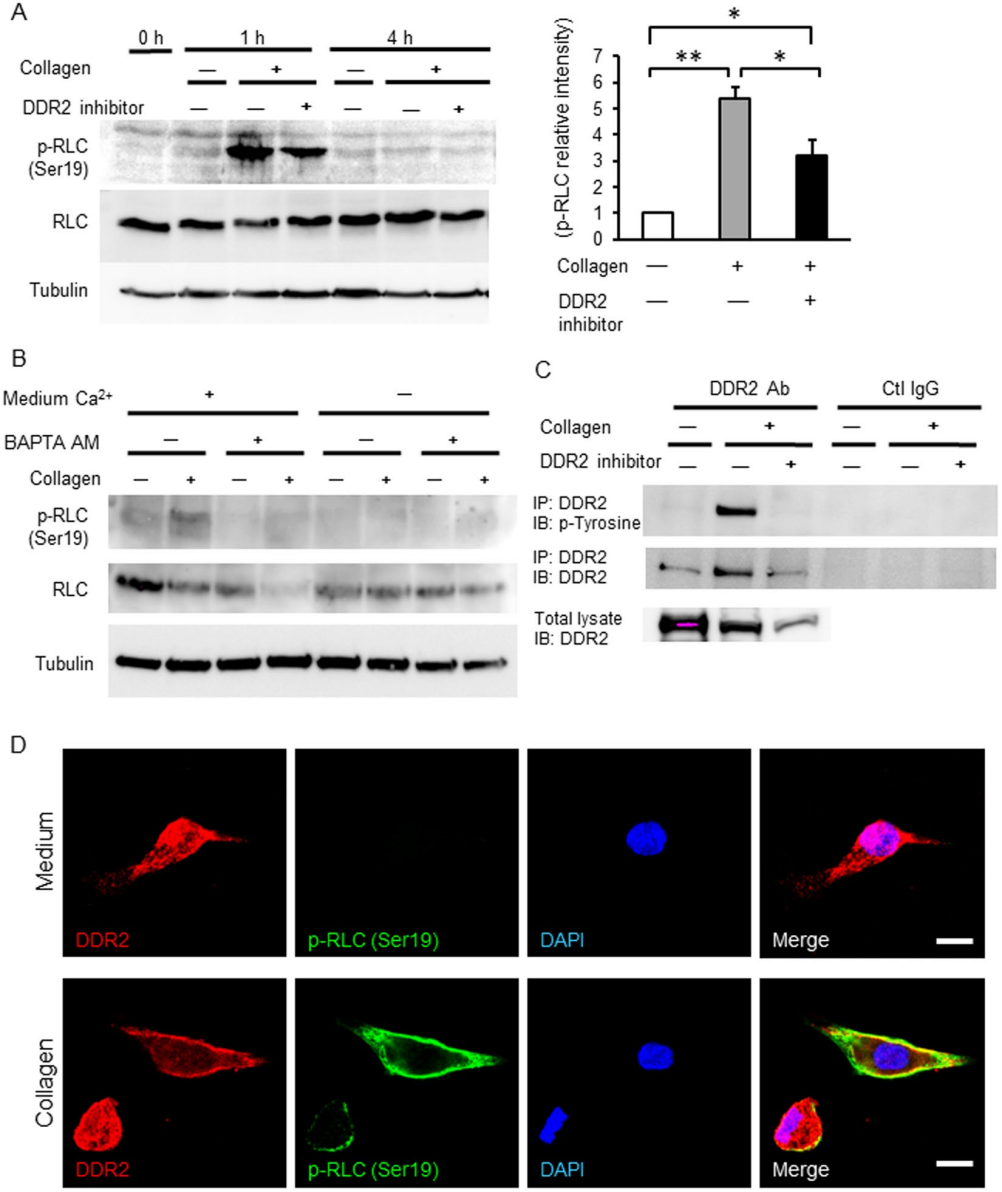
Collagen gel phosphorylated DDR2, a receptor tyrosine kinase, and activated myosin by phosphorylation of regulatory light chain. (**A**) Left panel: Immunoblots of phospho-myosin regulatory light chain (p-RLC), myosin regulatory light chain (RLC), and tubulin. Confluent primary human amnion mesenchymal cells were scratched, and then treated with collagen type 1 gel (2 mg/ml), collagen gel including DDR2 inhibitor (1 μM), or medium only as a control. After gel was solidified, medium containing DMSO or 1 μM of DDR2 inhibitor was added. Cells were incubated for 1 and 4 h. Right graph: Relative intensity of p-RLC at 1 h. Band intensity of p-RLC was compared with that of control (medium only, without collagen nor DDR2 inhibitor). Data was analyzed from 3 independent experiments. *P < 0.05, **P < 0.01, ANOVA. (**B**) Immunoblots of RLC after chelation of intracellular Ca^2+^ and extracellular. Confluent primary amnion mesenchymal cells were maintained in serum-free DMEM for 18 h and then incubated in DMEM or Ca^2+^-free DMEM for 6 h followed by pre-treatment with either DMSO or BAPTA-AM (5 µM) for 1 h. Thereafter, cells were covered by collagen type 1 gel or medium only for 1 h. (**C**) Immunoprecipitation of DDR2. Scratched primary human amnion mesenchymal cells were treated with medium, collagen gel, collagen gel including DDR2 inhibitor (1 μM) for 1 h. Lysates were immunoprecipitated with DDR2 antibody. Tyrosine phosphorylation of DDR2 immunoprecipitates was examined by immunoblotting. The membrane was reprobed with DDR2 antibody. (**D**) Immunofluorescence image of DDR2 and phospho-RLC. Primary human amnion mesenchymal cells were treated with medium or collagen for 1 h and stained for DDR2 (red), phosphor-RLC (p-RLC, green) and DAPI (blue). Bars, 10 μm.

Phosphorylation of myosin regulatory light chain (RLC) is a key factor in contraction of acto-myosin fibers and cell migration. Interaction of actin and myosin is regulated by Ca^2+^/calmodulin activation of myosin light chain kinase (MLCK) to phosphorylate serine 19 of RLC of myosin^8^. To test the effect of collagen type 1/DDR2 interactions on RLC phosphorylation in mesenchymal cells, cells were treated with type 1 collagen for 1 and 4 h ± the DDR2 inhibitor LCB03-0110 and immunoblot analysis was conducted. Collagen type 1 increased transient phosphorylation of RLC at 1 h returning to basal levels within 4 h (Fig. 3A). Inhibition of DDR2 decreased collagen-induced RLC phosphorylation at 1 h (Fig. 3A). Quantification of band intensity revealed 5.4-fold increase of p-RLC by collagen compared to medium (*P* < 0.01, n = 3 independent experiments), and DDR2 inhibitor with collagen decreased p-RLC by 40% (3.2-fold increase compared to medium) compared to collagen only (*P* < 0.05, n = 3).

Since activation of MLCK is Ca^2+^ -dependent, the effects of collagen-induced phosphorylation of RLC were determined in the presence or absence of Ca^2+^. Pretreatment of amnion mesenchymal cells with BAPTA-AM, a cell permeable intracellular Ca^2+^ chelator, and lack of extracellular Ca^2+^ in the media blocked collagen-induced phosphorylation of RLC (Fig. 3B).

DDR2 is a receptor tyrosine kinase, and maximal activation of DDR2 occurs several hours after stimulation with collagen^9^. Tyrosine-471 is a critical phosphotyrosine residue of DDR2^10^. In amnion mesenchymal cells, collagen phosphorylated tyrosine-471 of DDR2 and this phosphorylation was completely blocked by DDR2 inhibitor LCB03-0110 (Fig. 3C). Immunocytochemistry revealed that DDR2 was ubiquitously expressed in amnion mesenchymal cells throughout, and that collagen stimulation (i) relocalized DDR2 to the cell periphery (Fig. 3D, higher magnification, and S2D, lower magnification), (ii) increased phosphorylation of RLC, and (iii) localization of phosphorylated RLC and DDR2 overlapped at the cell periphery (Figs 3D and S2D). Together, these data indicate that collagen induces cell migration, DDR2 activation and highly localized Ca^2+^-dependent activation of myosin at the cell edge.

## Discussion

Here, we investigated the mechanisms of healing of sterile preterm premature rupture of the fetal membranes. Using a new model of pPROM in mice and primary human amnion cells in culture, we demonstrated that collagen type 1 increased healing of fetal membranes not only by likely scaffold formation but also stimulation of migration through activation of DDR2 receptors thereby activating myosin in amnion mesenchymal cells.

Two subtypes of DDR in mammals, DDR1 and DDR2 are widely expressed in fetal and adult tissues^9^ with DDR1 predominantly expressed in epithelial cells and DDR2 confined to mesenchymal cells^7^. Collagen-induced activation of DDRs is known to regulate cell adhesion, migration, proliferation, differentiation, and extracellular remodeling^9^. DDR2 is only activated by fibrillar collagens type I and type III resulting in phosphorylation of DDR tyrosine residues and subsequent activation of several downstream transcriptional factors. DDR1 is associated with non-muscle myosin IIA heavy chain upon ligand stimulation and promotes migration of NIH3T3 cells^11^. In fetal amnion mesenchymal cells, myosin regulatory chain was activated by DDR2 which was highly expressed in these cells. In addition to integrins and DDRs, other collagen receptors (glycoprotein VI and leucocyte-associated immunoglobulin-like receptor-1) are expressed in platelets, but not other mammalian cells.

Unexpectedly, M2 macrophages were entrapped within the collagen gel. We have already demonstrated that macrophages aggregate in the healing site of amnion in mouse pPROM model, which suggest their inevitable roles in the wound healing of amnion. Importantly, explant culture of traumatized human fetal membranes on collagen support did not show closure of the defect^12^. This indirectly suggests that fetal membrane is not able to heal autonomously, but it needs support of other cell types including macrophages. Therefore, we suggest that these trapped amniotic fluid macrophages augments healing of the fetal membranes.

The concept that collagen type 1 and matrix scaffolds regulate immune cell-mediated function is an exciting area of future research and suggests the potential usefulness of collagen for clinical application to pPROM. Although gelatinous at 4 °C and thereby easily injectable at the ruptured site, collagen gels solidify quickly at body temperature and forms a mechanical patch to barrier the rupture. Compared with sheets or solid material applications, host vs graft or graft vs host responses would be limited by eventual incorporation or dissolution of the native protein.

Various forms of biological materials have been used as a tissue sealant in animal models and clinical trials of human wounds including fibrin glue, platelet rich plasma, cryoprecipitates, fibrinogen, thrombin, polytetrafluoroethylene material, matrigel, and gelatin sponges and these trials are precisely reviewd by Devlieger^13^. Unfortunately, these trials were still pre-clinical research and not available in clinics. In addition, fetal fibronectin includes an extra-domain A (EDA) domain, which binds to Toll-like receptor 4, a receptor of innate immune system, and causes inflammatory responses in human amnion cells^14^. Thrombin and fibrinogen also activates TLR4^15,16^, which causes inflammation responses via NFkB pathway^17^. Hence, application of these TLR4-ligands has the potentialto induce intrauterine inflammation and preterm birth. Moreover, injection of platelet-rich plasma and platelets means blood transfusion and the a potential risk of infection. Therefore, the better and safer approaches for treatment of pPROM are warranted.

Using the rabbit as an animal model, Gratacos *et al*. showed that collagen gels with suture succeeded in rupture of fetal membrane induced by fetoscopy^18^. In addition, there is one case report in which a collagen graft was successful in human pPROM^19^. Data from this study provide mechanistic insights regarding the cellular biology of matricellular pharmacology on wound healing of the amnion. The amnion is the load-bearing structure of the fetal membranes and is thereby crucial for integrity and structural support of the fetus within the amniotic fluid.

Technical issues challenge the use of collagen gels for pPROM in women. Several trials have been unsuccessful using the transvaginal approach through the cervix to plug the site of rupture. Downward flow of amniotic fluid through the cervical canal, secretion of cervical glands, and the unsterile environment of the transvaginal approach are likely contributors to failure inhibiting effective plugging and healing. Further, precise detection of the rupture site was difficult. These studies suggest that sterile injection from the maternal abdomen with a thin needle and development of a modality to detect the rupture site in real-time may bring about successful interventions to treat PPROM. Results of the current investigation regarding the cellular and molecular mechanisms of collagen-induced healing of the membranes provide support to pursue solutions to these technical challengs.

To simplify the mechanism of healing by collagen, we used a sterile pPROM model. However in human, pPROM is caused by various causes such as infection, oxidative stresses, intrauterine bleeding, and mechanical stretch. The limitation of this study is that our model does not represent all causes of pPROM.

Although further research is necessary, this work provides basic insights for future clinical applications in healing the membranes primarily through augmentation of endogenous responses to wound healing but also stimulating migration of amnion mesenchymal cells.

## Materials and Methods

### Mouse model of sterile ruptured membrane

On 15 days post coitum (dpc), C57/Bl6 pregnant mice were anesthetized with isoflurane. A ventral incision was made and uterus and fetal membranes punctured with sterile 20 gauge needles (one puncture per gestational sac, Supplementary video 1). Immediately after rupture, 20 µl of PBS or rat Type I collagen gel (2 mg/ml, Cultrex 3-D Culture Matrix Rat Collagen I, #3447-020-01, Trevigen) was injected between fetal membranes and uterine lining of each fetus. In each uterus, half of gestational sacs were injected with PBS, and half were with collagen. Injection site was marked with 10% India Ink. After each procedure, the maternal abdomen was closed with 5-0 Polysorb. At the time of sampling, ruptured site was observed by microscopy. The ruptured edge of choriodecidua was visible after removal of myometrium (Fig. S1), and long axis of rupture was measured by a digital caliper. Edge of amnion was stained with black ink (Fig. S1) distinguishing it choriodecidua. Long axis of amnion rupture was then measured. When rupture of amnion is unclear, choriodecidua was slid with cotton swab, so the edge of ruptured amnion became visible. Closure was defined as an invisible hole under microscopy and the membrane continuous at the rupture site. Fetal membranes were collected after washing with PBS, quick frozen in liquid nitrogen, and stored at −80 °C. All procedures were approved by the Institutional Animal Care and Use Committee (IACUC) at the University of Texas Southwestern Medical Center.

### Quantitative real-time PCR

Total cellular RNA was extracted from cultured human amnion cells using RNAqueous-4PCR Kit (Ambion), according to the manufacturer’s protocol. Reverse transcription reactions were conducted using iScript cDNA Synthesis Kit (Biorad, #1708891) according to the manufacturer’s protocol. Quantitative RT-PCR was used to determine the relative levels of gene expression (Applied Biosystems, ABI 7900HT). Primer sequences for amplifications are as follows; β2 microglobulin forward: 5′-CGCTCCGTGGCCTTAGC-3′, reverse: 5′-AATCTTTGGAGTACGCTGGATAGC-3′. DDR1 forward: 5′-CCACCATCAGCTACCAAATGC-3′, DDR1 reverse: 5′-ACAAAGTTGAGTGTGGCCAGATAG-3′, DDR2 forward: 5′-TGTACGCACTGTCAGTTACACCAA-3′, and DDR2 reverse: 5′-CAGTTTCGTGTGGCCAGATCT-3′. SYBR Green to detect amplification and gene expression was normalized to that of indicated housekeeping gene.

### Isolation and culture of amnion epithelial and mesenchymal cells

Fetal membranes from term placentas of uncomplicated cesarean section were obtained through a protocol approved by the Institutional Review Board of the University of TX Southwestern Medical Center and all experiments were performed in accordance with relevant guidelines and regulations. Separation and isolation of amnion epithelial and mesenchymal cells were performed as previously described^20^. Reflected amnion tissue was separated by blunt dissection and then minced. Tissue and 150 ml of Eagles’s minimum essential medium (EMEM, #M0725, Sigma) containing 1 g of trypsin (Trypsin 1:250, #27250-018, Gibco) were stirred for 30 min. The mixture was poured into a sieve (CD1 Screen Cup, #S1145, with 40 mesh screen, #S0770, Sigma-Aldrich) to separate digested from remaining tissue. Thereafter, the medium was discarded, and tissue minced again and incubated at 37 °C in EMEM with trypsin (150 ml) for 30 min with stirring. Then, the mixture was poured over the sieve to separate dispersed amnion epithelial cells from tissue pieces. The remaining tissue pieces were minced, incubated again with trypsin (150 ml) for 30 min, and filtered through the sieve to remove epithelial cells. Mesenchymal cells were isolated from the remaining tissue. Tissue pieces were placed in 150 ml of EMEM that contained collagenase (0.75 mg/ml, Collagenase B, #11088831001, Roche) and DNase I (0.075 mg/ml, #10104159001, Roche) and were incubated at 37 °C for 30 min with stirring. The dispersed mesenchymal cells were collected by filtration of the mixture through sieve and centrifugation. Isolated amnion epithelial and mesenchymal cells were suspended in DMEM/F12 (D8437, Sigma) with 10% fetal bovine serum and 1% antibiotic-antimycotic solution (Antifungal 100X, #15240-062, Gibco). Cells were plated in plastic culture dishes, maintained at 37 °C in a humidified atmosphere of 5% CO_2_ in air, and allowed to replicate in monolayer to confluence.

### Immunofluorescent staining

Mouse uterus was fixed in 4% paraformaldehyde overnight and kept in 50% ethanol until paraffin embedding. After sections (5 μm) were deparaffinized, antigen retrieval was performed by incubation with proteinase K (P8107S, New England Biolab, working concentration, 0.6 units/ml) for 1 minute at 37 °C. For antigen retrieval of E-cadherin, and vimentin, sections were boiled in 10 mM sodium citrate buffer (pH 6.0) by microwave for 20 min, and then cooled for 30 minutes at room temperature. After antigen retrieval, sections were preincubated with 10% normal goat serum (50062Z, Life Technologies) with 0.3% Triton X-100 for 30 minutes at room temperature. Subsequently, tissue sections were incubated with primary antibody in PBS with 1% BSA and 0.3% Triton X-100 at 4 °C overnight. Primary antibodies used and concentration were as follows: F4/80 (clone BM8, eBioscience, #14-4801, 1:50), Vimentin (D21H3, Cell Signaling, #5741, 1:100), E-cadherin (4A2, Cell Signaling, #14472, 1:50), and Arg1 (Arginase-1, D4E3M, Cell Signaling, #93668, 1:100). Thereafter, sections were incubated with Alexa Fluor 488, 549, or 594–conjugated secondary antibodies (Ig, heavy and light chains, Invitrogen, 1:500 dilution) in 10% normal goat serum for 1 h at room temperature. After that, slides were mounted with Prolong Gold Antifade Reagent with 4′,6-Diamidino-2-Phenylindole (DAPI, P36935, Molecular Probes). Images were taken by Leica SP5 confocal microscopy. Image J software was used to generate individual images.

### Wound healing scratch assay

Primary human amnion epithelial or mesenchymal cells were harvested in 12-well plate. At confluency, the center of each well was scratched with a 10 µl tip (epithelial cells) or a 200 µl tip (mesenchymal cells). The width of wound was measured microscopically with 3 points in each field. Three different fields were selected in each well, and width was averaged. Percent closure was calculated by dividing the average width of the indicated time by the width of 0 h.

### Immunocytochemistry

Primary human amnion epithelial or mesenchymal cells were grown in 8-well chamber slide. Cells fixed in 4% paraformaldehyde for 10 minutes. After washing with PBS, slides were incubated with 10% normal goat serum for 30 minutes at room temperature. Thereafter, slides were incubated with primary antibodies overnight at 4 °C. Primary antibodies used and concentration was as follows: DDR2 (#290814, R&D systems, 10 µg/ml), and Phospho-Myosin Light Chain 2 (Ser19, Cell Signaling, #3671, 1:50). After incubation with secondary antibody (Alexa Fluor 488, 549, or 594, Invitrogen, 1:500 dilution), slides were mounted with Prolong Gold DAPI. Images were taken by Leica SP5 confocal microscopy, and generated by Image J software.

### Collagen gel preparation and scratch assay in primary human amnion cells

Type-1 collagen gel (Cultrex 3-D Culture Matrix Rat Collagen I, #3447-020-01, Trevigen) was prepared according to the manufacture’s protocol. Briefly, collagen I was added and mixed in appropriate volumes of 10X PBS, 1 N NaOH and dH2O in sterile tubes. Blocking antibodies or inhibitors were used as follows: anti-integrin α1 antibody (clone FB12, #MAB1973Z, Millipore), anti-integrin α2 antibody (clone P1E6, #MAB1950Z, Millipore), anti-integrin β1 antibody (clone 6S6, #MAB2253Z, Millipore), Mouse IgG1 control antibody (Clone 107.3, #554721, BD Bioscience), DDR1 inhibitor (DDR1-IN-1, #5077, Tocris), DDR2 inhibitor (LCB 03-0110, #5592, Tocris), integrin α1β1 inhibitor (Obtustatin, #4664, Tocris) and integrin α2β1 inhibitor (BTT 3033, #4724, Tocris). These antibodies or inhibitors were incorporated into gel at indicated concentration. Thereafter, collagen gel was placed to the dishes or plates, where confluent primary amnion cells have been scratched. Dishes or plates were incubated at 37 °C for 20 minutes to promote gel formation. After gel was solidified, medium was gently added with drop-wise in the wells or dishes. Antibodies and inhibitors were also added to the medium at indicated concentration.

### siRNA transfection

siRNA experiments were performed according to manufacturer’s instruction. Briefly, primary amnion human cells were passaged to 48-well plate at the concentration of 0.5 × 10^5^ cells/well. Next day, after changing medium to Optimem I (#11058-021, Gibco), siRNA-lipid complex was added to the cells using Lipofectamine RNAiMAX Reagent (#13778, Invitrogen), Allstars Neg. Control siRNA (#1027281, Qiagen) as negative control siRNA, Hs DDR2 4 FlexiTube siRNA (as si-DDR2-1, #SI00085274, Qiagen), Hs DDR2 2 FlexiTube siRNA (as si-DDR2-2, #SI00085260, Qiagen). After 24 h of transfection, scratch assay was started covering the cells with 2 mg/ml of collagen I gel.

### Immunoblots

Confluent primary human amnion cells were scratched with 10 µl tip for 32 times (10 sagittal lines, 10 horizontal, and 6 + 6 oblique lines), and then covered with collagen gel. After treatment, cells were lysed in RIPA buffer (0.15 M NaCl, 1% NP40, 0.5% Sodium deoxycholate, 0.1% SDS, and 50 mM Tris) containing protease inhibitor cocktail (Complete Mini, Roche) and phosphatase inhibitor cocktail (PhosSTOP, Roche). The collected samples were incubated overnight at 4 °C under gentle rotary agitation. Next day, the samples were centrifuged at 10,000 G for 20 min and the supernatant were used for immunoblots. After SDS-PAGE, the membranes were blocked with 5% BSA or nonfat powdered milk for 1 h at room temperature. A membrane was incubated with primary antibodies overnight at 4 °C as follows: Phospho-Myosin Light Chain 2 (or p-RLC, Ser19, Cell Signaling, #3671, 1:1000), myosin light chain 2 (or RLC, D18E2, #8505, Cell Signaling, 1:1000), phosphotyrosine (Clone 4G10, #05-321, Millipore, 1:1000), DDR2 (#290814, R&D systems, 1:1000), tubulin (#2148, Cell Signaling, 1:1000). Thereafter, blots were incubated with secondary antibody (Goat Anti-Rabbit IgG-HRP Conjugate, #170-6515, Biorad, 1:10000 or Goat Anti-Mouse IgG-HRP Conjugate, #170-6516, Biorad, 1:10,000) at room temperature for 1 h. The signal was detected by chemiluminescence (Pierce ECL Plus Western Blotting Substrate, #32132, Thermo Scientific). For quantification of band intensity, Image J software was used.

### Immunoprecipitation

60 µg of total protein extracted was added into of RIPA buffer containing protease inhibitor and phosphatase inhibitor cocktail, and total volume was adjusted to 200 µl. Protein was incubated with 2 µl of anti-DDR2 antibody (#12133, Cell Signaling) or Normal Rabbit IgG (#2729, Cell Signaling) overnight under rotary agitation (final concentration of antibody was 1:100). Next day, 70 µl of protein A/G beads (Pierce Protein AG Plus Agarose, #20423, Thermo Scientific) were added to the mixture of protein and antibody, incubated for 2 h at 4 °C under rotary agitation. After wash with RIPA buffer three times, proteins were eluted with 30 µl of 2X Laemmli SDS sample buffer including 10% beta-mercapthoetahnol at 95 °C for 5 minutes and separated by SDS-PAGE.

### Statistical Analysis

Values were expressed as means ± SD *in vitro* experiments or means ± SEM in mice experiments. Data were analyzed by unpaired Student’s *t*- test unless indicated otherwise. *P*-values less than 0.05 were regarded as statistically significant. In Figs 2C–E and 3A, one-way ANOVA was conducted. For the comparison of complete closure of amnion and choriodecidua (Fig. 1H), chi-square test was utilized.

## Electronic supplementary material

Supplementary information
