# Supplementary material for: Collagen Type 1 Accelerates Healing of Ruptured Fetal Membranes
Source: Sci Rep. 2018 Jan 12;8:696. doi: 10.1038/s41598-017-18787-9 (PMC5766504; doi:10.1038/s41598-017-18787-9)
Supplement: Supplementary file 1 — Supplementary information [file 41598_2017_18787_MOESM1_ESM.pdf]

Collagen Type 1 Accelerates Healing of Ruptured Fetal Membranes

Haruta Mogami, Annavarapu Hari Kishore, and R. Ann Word

Figure S1

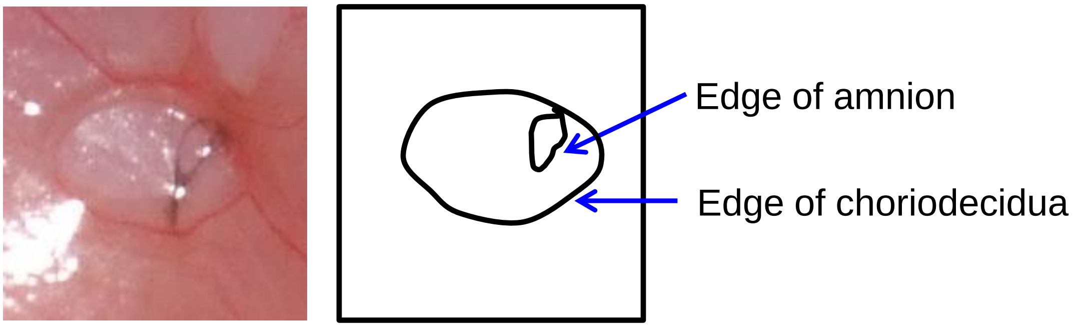

Figure S1. Measurement of rupture size. Ruptured fetal membrane after removal of myometrium and its scheme. Note that edge of ruptured amnion was stained with black ink.

Figure S2

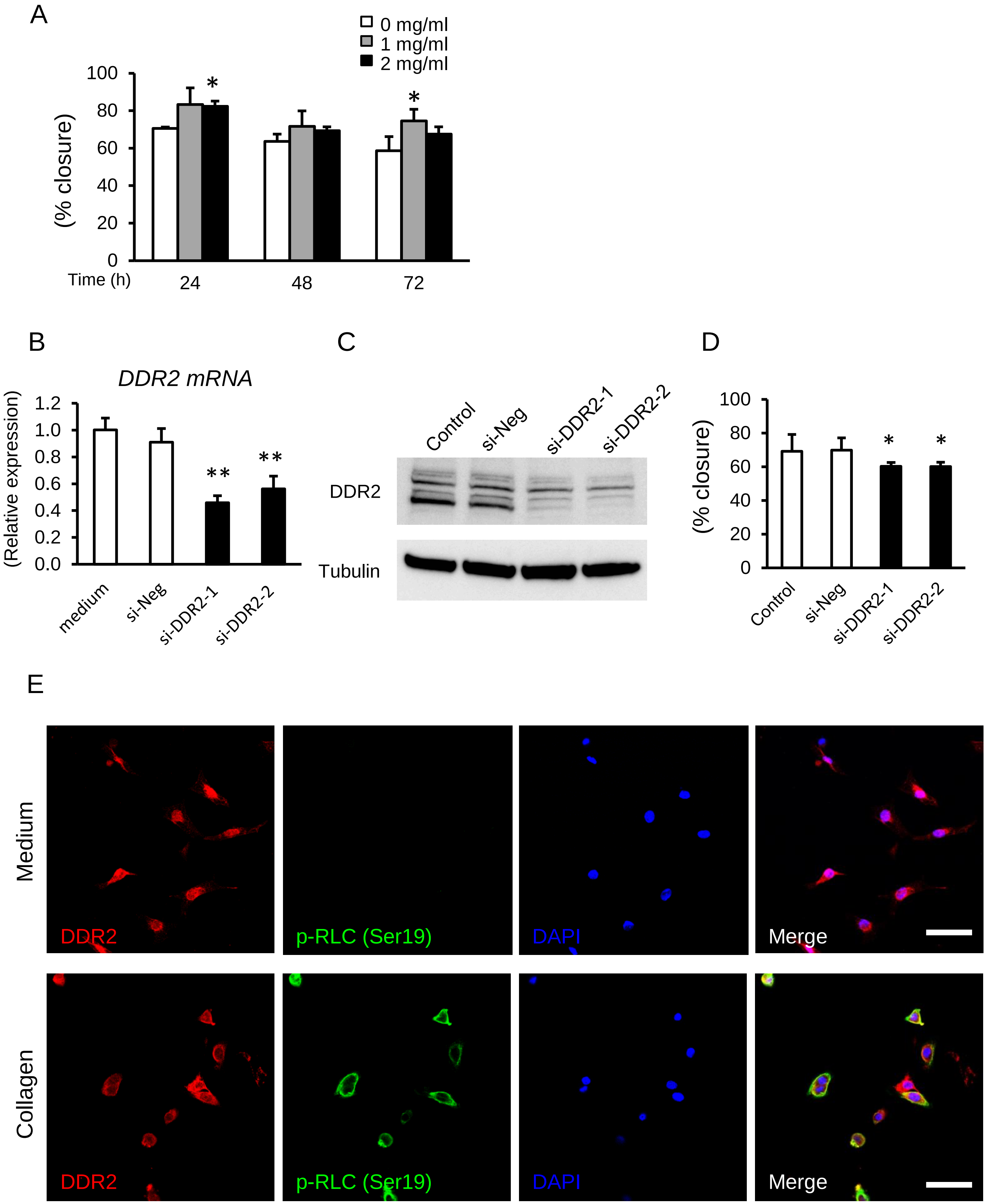
A
0 mg/ml
1 mg/ml
2 mg/ml
(% closure)
100
80
60
40
20
0
Time (h)
24
48
72
*
*
B
DDR2 mRNA
(Relative expression)
1.2
1.0
0.8
0.6
0.4
0.2
0.0
**
**
medium
si-Neg
si-DDR2-1
si-DDR2-2
C
Control
si-Neg
si-DDR2-1
si-DDR2-2
DDR2
Tubulin
D
(% closure)
100
80
60
40
20
0
*
*
Control
si-Neg
si-DDR2-1
si-DDR2-2
E
Medium
Collagen
DDR2
p-RLC (Ser19)
DAPI
Merge

Figure S2. The effect of collagen gel on migration of human amnion cells. (A) Wound healing scratch assay of primary human epithelial cells with collagen gel. Confluent epithelial cells were scratched and treated with different concentration of collagen type 1 gel. Percent closure of scratched area was calculated relative to the width at 0 h. Error bars represent SD. n=3 in each group. *$P < 0.05$, **$P < 0.01$. **(B-D)** Knockdown of DDR2 by siRNA. **(B)** *DDR2* mRNA and **(C)** protein expression after transfection with control (medium only), negative control siRNA (si-Neg) or 2 different si-DDR2 RNAs for 48 h. **(D)** Scratch assay of primary human amnion mesenchymal cells transfected by si-DDR2 RNAs. Percent closure after 24 h of collagen treatment. Error bars represent SD. **(B)** n=3 and **(D)** n=6 in each group. *$P < 0.05$ and **$P < 0.01$ compared to si-Neg. **(E)** Immunofluorescence image of DDR2 and phospho-RLC by confocal microscopy (lower magnification of Figure 3D). Primary human amnion mesenchymal cells were treated with medium or collagen for 1 h and stained for DDR2 (red), phospho-RLC (p-RLC, green) and DAPI (blue). Bars, 50 μm.
